# Supplementary material for: Photo-thermoresponsive polypyrrole-crosslinked single-chain nanoparticles for photothermal therapy
Source: Commun Chem. 2025 Apr 25;8:124. doi: 10.1038/s42004-025-01518-x (PMC12032120; doi:10.1038/s42004-025-01518-x)
Supplement: Supplementary file 2 — Supporting Information [file 42004_2025_1518_MOESM2_ESM.pdf]

Photo-Thermoresponsive Polypyrrole-Crosslinked Single-Chain Nanoparticles for Photothermal Therapy

Justus F. Thümmler, Farzin G. Golmohamadi, Daniel Schöffmann, Jan Laufer, Henrike Lucas, Julia Kollan, Karsten Mäder, Wolfgang H. Binder*

Analytical Methods 2
Syntheses 6
NMR Spectra 9
Supporting Figures and Tables 17

Analytical Methods

NMR spectra were measured on an Agilent Technologies 400 MHz VNMRS and 500 MHz DD2 at 27°C. Chemical shifts (δ) are reported in ppm and referred to the solvent residual signal ($CDCl_3$ 7.26 ppm for ^1H and 77.0 ppm for ^{13}C, D_2O 4.66 ppm for ^1H).

DOSY measurements were done on an Agilent VNMR DD2 500 MHz (sfrq = 499.727 MHz). The experiment was performed under OpenVnmrJ 1.1 and equipped with a 5 mm PFG One NMR probe, z-gradient and temperature unit (27°C). Diffusion ordered NMR data were acquired by means of the Agilent pulse program DgcsteSL_cc using a stimulated echo with self-compensating gradient schemes and conventional compensation. The length of the gradient pulse was set to 3.0 ms for ^1H in combination with a diffusion period of 300 ms (D_2O). Data were systematically accumulated by linearly varying the diffusion encoding gradients over a range from 2% to 95% for 64 gradient increment values.

SEC measurements were performed at 30°C on a Viscotek GPCmax VE 2001 from Viscotek™ applying a CLM3008 precolumn and a CLM3008 main column. As solvent THF was used and the sample concentration was adjusted to 3 mg ml^{-1} while applying a flow rate of 1 ml min^{-1}. For determination of the molecular weights the refractive index of the investigated sample was detected with a VE 3580 RI detector of Viscotek™. External calibration was done using poly(styrene) standards (purchased form PSS) with a molecular weight range from 1050 to 115000 g mol^{-1}.

AFM measurements were performed using a nanosurf CoreAFM with Tap190Al-G cantilevers in the phase-contrast mode. The samples were dissolved in milipore water at concentrations of 50 µg/mL. The solutions were dropped on mica surfaces, incubated for 30 seconds and removed with precision wipes before measurements. Data analysis was performed using Gwyddion 2.61 (freeware, http://gwyddion.net/).

Turbidimetry measurements were performed on a JASCO J-1500 with a PTC-510 cell holder. Samples were measured in a Helma analytics quartz glass cuvettes (d = 1 mm) at concentrations of 1 mg/mL in water, and were heated from 30°C to 90°C with a heating rate of 1 K/min. Transmittance was measured at a wavelength of 400 nm. The temperature at 90% of the normalized transmittance was taken as the cloud point temperature T_{cp}.

Dynamic Light Scattering was performed using the Zetasizer Pro from Anton Paar. A solution of $SCNP_2$ in PBS was set to a concentration of 2 mg/mL and then diluted with PBS and with BSA (10%) to reach a final SCNP concentration of 1 mg/mL. The mixtures were equilibrated at room temperature for 24h before the measurements were performed. The determined hydrodynamic diameter were calculated from the number average distributions.

VIS/NIR-absorption measurements were performed on a Perkin Elmer LAMBDA 365 UV/Vis Spectrophotometer using Helma analytics quartz glass cuvettes (*d* = 10 mm).

Laser-induced photothermal temperature variations were measured at concentrations from 0.1 mg/mL to 2 mg/mL in water. The samples were filled in a quartz glass cuvette (*d* = 10 mm) and illuminated with laser pulses (SpitLight EVO III, pulse length = 5 ns, repetition rate = 100 Hz, λ = 815 nm, power = 5.5 W/cm²) which were guided on the cuvette surface with a CeramOptec fiber bundle. Temperature was measured simultaneously with a thermocouple type K (Chromel/Alumel) connected to a NiCr-Ni-adapter S, type K. After 10 min of illumination the samples were cooled down for 15 min and illuminated again. The solutions were stirred continuously during the experiment.

96-well plate illuminations were done using the Lumidox II 96-Well LED Array 730-IR from Analytica Sales with a diffuse mat surface and a solid base with thermal transfer deck. The emitted peak wavelength was determined to be 721.99 nm. The apparatus was cooled using a thermostat set to 15°C. The radiant fluxes were set to 190 mW (Irradiance 559 mW/cm²) or 285 mW (838 mW/cm²).

Thermal Imaging was performed using the FLIR T650sc.

Cell viabilities of the SCNP formulations were tested using a Resazurin reduction 96 well assay on DLD-1 and 3T3 cells. Two time points (24 and 96 h) were analyzed. DLD-1 were cultured in RPMI + 10% FCS + 1% penicillin-streptomycin at 37°C under 5% CO_2 in a standard cell culture incubator. The medium for 3T3 was DMEM (high glucose) + 10% FCS + 1% penicillin-streptomycin additionally supplemented with sodium pyruvate (final concentration (f.c.) 1 mM). Both cell lines were seeded in white 96 well plates with transparent bottoms. Seeding numbers were as follows: For 24 h 5,000 cells/100 µL and 10,000 cells/100 µL and for 96 h 1500 cells/100 µL and 5,000 cells/100 µL were used for DLD-1 and 3T3, respectively. On the day after seeding, SCNP formulations (serial dilution in corresponding cell culture medium) were added in increasing concentrations from 0.0001 mg/ml to 1.0 mg/mL f.c.. Pure cell culture medium served as negative control (= 100% viability) and 0.025% Triton X100 was used as positive control (= 0% viability). For background correction, one column was filled with cell culture medium without any cells. All varying concentrations of the assay were performed as octuplicates (n = 8). After incubating for 24 or 96 h, resp., 20 µL Resazurin stock solution (440 µM in PBS, f.c. 44 µM) was added to each well and the mixture was incubated for 2 h at 37°C under 5% CO_2. Then, fluorescence intensity was determined with the Cytation™ 5 imaging reader (BioTek Instruments) equipped with a BP531(20) filter for excitation and a BP593(20) filter for emission. Cell viability was expressed as a percentage of the negative controls (untreated cells) after subtraction of the blank. The assay was performed three times and then, mean and S.D. were calculated and plotted for data evaluation (n = 3).

Photothermal cell viabilities were also measured by the Resazurin reduction assay (see also paragraph Cell viabilities). One day after seeding, SCNP solutions were added to the wells leading to the three different f.c. 0.001 mg/mL (low), 0.01 mg/mL (medium) and 0.1 mg/mL (high). Pure cell culture medium served as negative control (= 100% viability) and 0.025% Triton X100 was used as positive control (= 0% viability). The sole illumination effect was tested using cells in 96 well plates without the adding of any SCNP. In that case, volume was filled up with the corresponding cell culture medium. For background correction, one column was filled with cell culture medium without any cells. All varying concentrations of the assay were performed as octuplicates (n = 8). Then, plates were incubated for 2 h under standard conditions (37°C, 5% CO_2) to enable particle ad- and/or absorption on or into the cells, respectively. Afterwards, illumination was performed on the Lumidox II 96-Well LED Array 730-IR (see paragraph 96 well plate illuminations) for 15 min at the two different outputs 190 mW or 285 mW, respectively. After incubating for additional 96 h, 20 µL Resazurin stock solution (440 µM in PBS, f.c. 44 µM) was added to each well and the mixture was incubated again for 2 h at 37°C under 5% CO_2. Then, fluorescence intensity was determined with the Cytation™ 5 imaging reader (BioTek Instruments) equipped with a BP531(20) filter for excitation and a BP593(20) filter for emission. Cell viability was expressed as a percentage of the negative controls (untreated cells) after subtraction of the blank. The assay was performed three times and then, mean and S.D. were calculated and plotted for data evaluation (n = 3).

Photoacoustic spectra and images were measured using the setup shown in Figure S14. PA spectroscopy measurements were conducted using a custom-built, calibrated PA spectrometer to generate and detect PA waves. The experimental setup included a cuvette containing purified $SCNP_{10}$ solutions, designed with an approximate pathlength of 5.3 mm and an 18 mm diameter to accommodate small sample volumes. This cuvette was immersed in a water bath maintained at room temperature. PA waves were detected using a planar PVDF transducer (19 mm diameter) with a -6 dB bandwidth of 20 MHz and a cutoff frequency of 37 MHz (Precision Acoustics). The signals were amplified using a 20 dB voltage amplifier (Femto Messtechnik GmbH) and digitized with a National Instruments data acquisition card. PA measurements were performed across $SCNP_{10}$ concentrations ranging from 0.1 mg/ml to 2 mg/ml. For imaging, a custom PA tomography system (PhotoSound Technologies) was employed to obtain 2D images of a phantom. The phantom comprised two translucent silicone tubes (silastic, 1.47 mm inner diameter, 1.96 mm outer diameter) filled with $SCNP_{10}$, along with a $CuSO_4$ solution serving as a negative control, all submerged in a water bath at room temperature. PA signals were captured using a focused ring array ultrasound transducer (512 elements, Imasonic) with a center frequency of 5.5 MHz and a 55% bandwidth. Data acquisition was managed via custom LabView software. Imaging was performed with optical

pulses delivered through a 1-to-8 fiber bundle to ensure uniform light distribution around the phantom. PA signals were reconstructed using an in-house backprojection-based algorithm.

Syntheses

All chemicals were purchased from Sigma Aldrich, except for potassium hydroxide (Carl Roth), 6-chlorhexanol (TCI), and triethylamine (Alfa Aesar). Before use, pyrrole was freshly distilled, and oligo (ethylene glycol) methyl ether methacrylate (OEGMA, M_n = 300 Da) was passed through a basic AlO_x-column to remove the stabilizer.

Synthesis of 6-pyrrolylhexanol

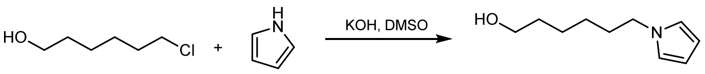

Pyrrole (60 mmol, 4.02 g, 4.15 mL) was added to a dispersion of KOH (90 mmol, 4.5 g) in dry DMSO (100 mL). 6-Chlorohexanol (20 mmol, 2.73 g, 2.67 mL) was dissolved in 50 mL dry DMSO and added dropwise to the stirring KOH dispersion, and was stirred at room temperature for 18 hours. The solution was filtrated and diluted with 250 mL water. The product was extracted with 3 x 50 mL diethyl ether and dried over Na_2SO_4. The solvent was removed under reduced pressure. The crude product was purified *via* column chromatography (cyclohexane : ethyl acetate 2:1, R_f = 0.23) to get the product as a yellow liquid. Yield: 2.15 g, 64%. ^{1}H-NMR ($CDCl_3$, 400 MHz, δ in ppm): 6.67 (2H, m, CH_{Ar}N), 6.17, (2H, m, CH_{Ar}), 3.89 (2H, t, ^3J = 7.1 Hz, CH_2N), 3.59 (2H, t, ^3J = 6.6 Hz, CH_2O), 2.41 (1H, broad, O*H*), 1.80 (2H, m, CH_2-CH$_2$N), 1.56 (2H, m, CH_2-CH$_2$O), 1.36 (4H, m, CH$_2$CH_2-CH_2-CH$_2$). ^{13}C{^{1}H}-NMR ($CDCl_3$, 100 MHz, δ in ppm): 120.5 (C_{Ar}), 107.8 (C_{Ar}), 62.5 (*C*H$_2$O), 49.5 (*C*H$_2$N), 32.5 (*C*H$_2$CH$_2$O), 31.5 (*C*H$_2$CH$_2$N), 26.6 (*C*H$_2$), 25.4 (*C*H$_2$).

Synthesis of 6-pyrrolylhexyl methacrylate ***(PyrHexMA)***

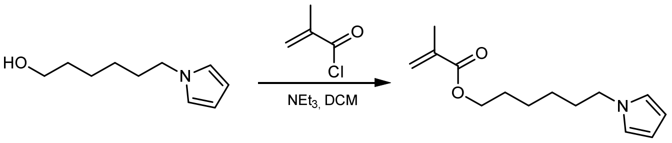

1 (12 mmol, 2g) and triethylamine (24 mmol, 2.42 g, 3.33 mL) were dissolved in 20 mL dry DCM, stirred for 1 hour and then placed in an ice bath. Methacryloyl chloride (18 mmol, 1.88 g, 1.73 mL) was dissolved 5 mL dry DCM and added dropwise to the cooled solution. The reaction mixture was stirred at 0°C for 1 hour and then at room temperature for 18 hours. The solution was filtrated, washed with water (3 x 50 mL) and brine (50 mL) and dried over Na_2SO_4. The solvent was removed under reduced pressure. The crude product was purified *via* column chromatography (cyclohexane : ethyl acetate 2:1, R_f = 0.59) to get the product as a yellow liquid. Yield: 942 mg, 33%. ^{1}H-NMR ($CDCl_3$, 400 MHz, δ in ppm): 6.64 (2H, m, CH_{Ar}N), 6.13, (2H, m, CH_{Ar}), 6.09 (1H, dq, ^2J = 1.9 Hz, ^3J = 1.0 Hz, =C*H*), 5.55 (1H, dq, ^2J = 1.9 Hz,

^3J = 1.6 Hz, =C*H*), 4.13 (2H, t, ^3J = 6.6 Hz, C*H₂*O), 3.87 (2H, t, ^3J = 7.1 Hz, C*H₂*N), 1.94 (3H, dd, ^3J = 1.6, 1.0 Hz, C*H₃*COO), 1.78 (2H, m, C*H₂*-CH₂N), 1.67 (2H, m, C*H₂*-CH₂O), 1.35 (4H, m, CH₂C*H₂*-C*H₂*-CH₂). ^{13}C{^{1}H}-NMR ($CDCl_3$, 100 MHz, δ in ppm): 167.4 (*C*=O), 136.5 (CH_3*C*=CH_2), 125.2 (*C*H₂=C) 120.4 (C_{Ar}), 107.9 (C_{Ar}), 64.5 (*C*H₂O), 49.5 (*C*H₂N), 31.5 (*C*H₂CH₂N), 28.5 (*C*H₂CH₂O), 26.4 (*C*H₂), 25.6 (*C*H₂), 18.3 (*C*H₃).

Synthesis of poly (oligo (ethylene glycol) methacrylate)-co-(6-pyrrolylhexyl methacrylate) ***(Precursor Polymer)***

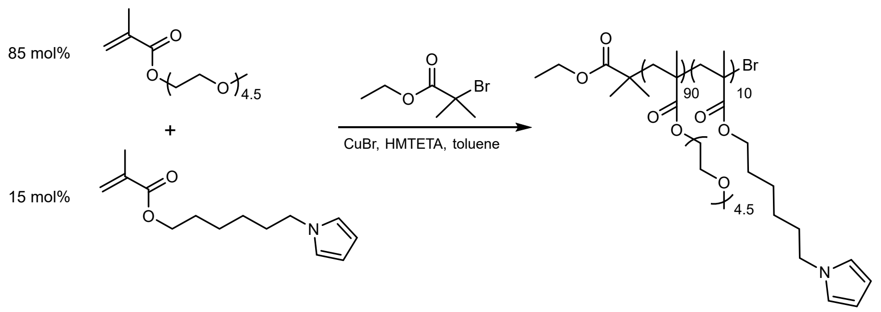

OEGMA (2.92×10^{-3} mol, 877.2 mg), PyrHexMa (5.16×10^{-4} mol, 121.43 mg), ethyl α-bromoisobutyrate (1.72×10^{-5} mol, 3.35 mg, 2.48 µL), and hexamethyltriethylenetetramine (3.42×10^{-5} mol, 7.88 mg, 9.30 µL) were dissolved in toluene. The mixture was degassed by five freeze-pump-thaw cycles and transferred into a glovebox. CuBr (3.42×10^{-5} mol, 7.64 mg) was added and the mixture was heated to 70°C for 72 h. The product solution was diluted with 5 mL THF, passed through silica, and precipitated into cyclohexane/diethyl ether (2:1). Yield: 561 mg, 56%. GPC (THF): M_n = 25.0 kDa, M_w = 36.8 kDa, $Đ$ = 1.47. ^{1}H-NMR ($CDCl_3$, 400 MHz, δ in ppm): 6.63 (CH_{Ar}N), 6.08 (CH_{Ar}), 4.25-3.94 (COOC*H₂*-OEG), 3.87 (COOC*H₂*-HexPyr) 3.82-3.40 (OC*H₂*C*H₂*O), 3.35 (OC*H₃*), 2.10-1.65 (C*H₂*), 1.14-0.62 (C*H₃*). ^{13}C{^{1}H}-NMR ($CDCl_3$, 100 MHz, δ in ppm): 120.4 (C_{Ar}), 107.8 (C_{Ar}).

General procedure for the synthesis of poly pyrrole crosslinked SCNPs ***($SCNP_x$)***

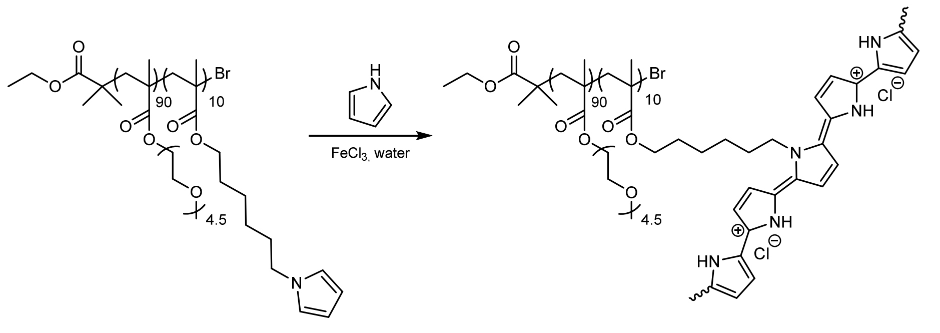

$FeCl_3$ (1.87×10^{-2} mol, 3.03 g) was dissolved in 100 mL degassed water and stirred vigorously. The precursor polymer (100 mg, 3.4×10^{-5} mol pyrrole) and additional pyrrole (0 eq., 2 eq (6.8×10^{-3} mol), 5 eq. (1.7×10^{-4} mol), and 10 eq. (3.4×10^{-4} mol)) were dissolved in 10 mL degassed water and added to the $FeCl_3$ solution using a syringe pump (0.5 mL/h). After the

complete addition, the solution was kept stirring for one additional hour. The product was extracted with Σ 100 mL DCM, dried with Na_2SO_4, passed through silica and dried under reduced pressure to get the brown-to-black product. Yield: 58%. ^{1}H-NMR ($CDCl_3$, 400 MHz, δ in ppm): 8.07 (CH_{Ar}), 6.64 (CH_{Ar}N), 6.09 (CH_{Ar}), 4.16-3.96 (COOCH_2-OEG), 3.87 (COOCH_2-HexPyr) 3.82-3.40 (OCH_2CH_2O), 3.35 (OCH_3), 2.10-1.52 (CH_2), 1.14-0.62 (CH_3). ^{13}C{^{1}H}-NMR ($CDCl_3$, 125 MHz, δ in ppm): 177.1 (*C*=O), 129.5 (C_{Ar}), 120.4 (C_{Ar}).

NMR Spectra

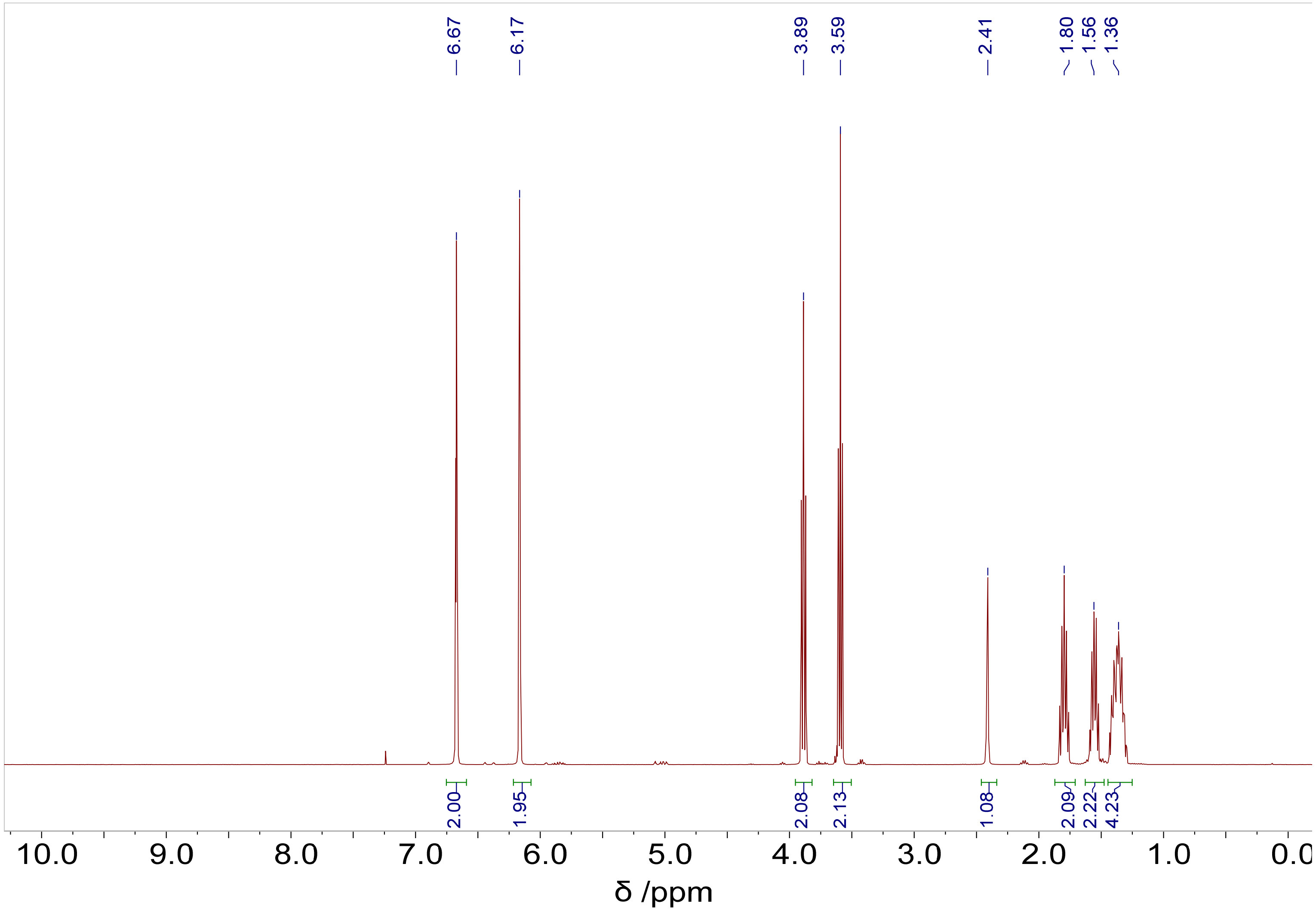

Figure S1. ^{1}H-NMR spectrum of 6-pyrrolylhexanol in $CDCl_3$.

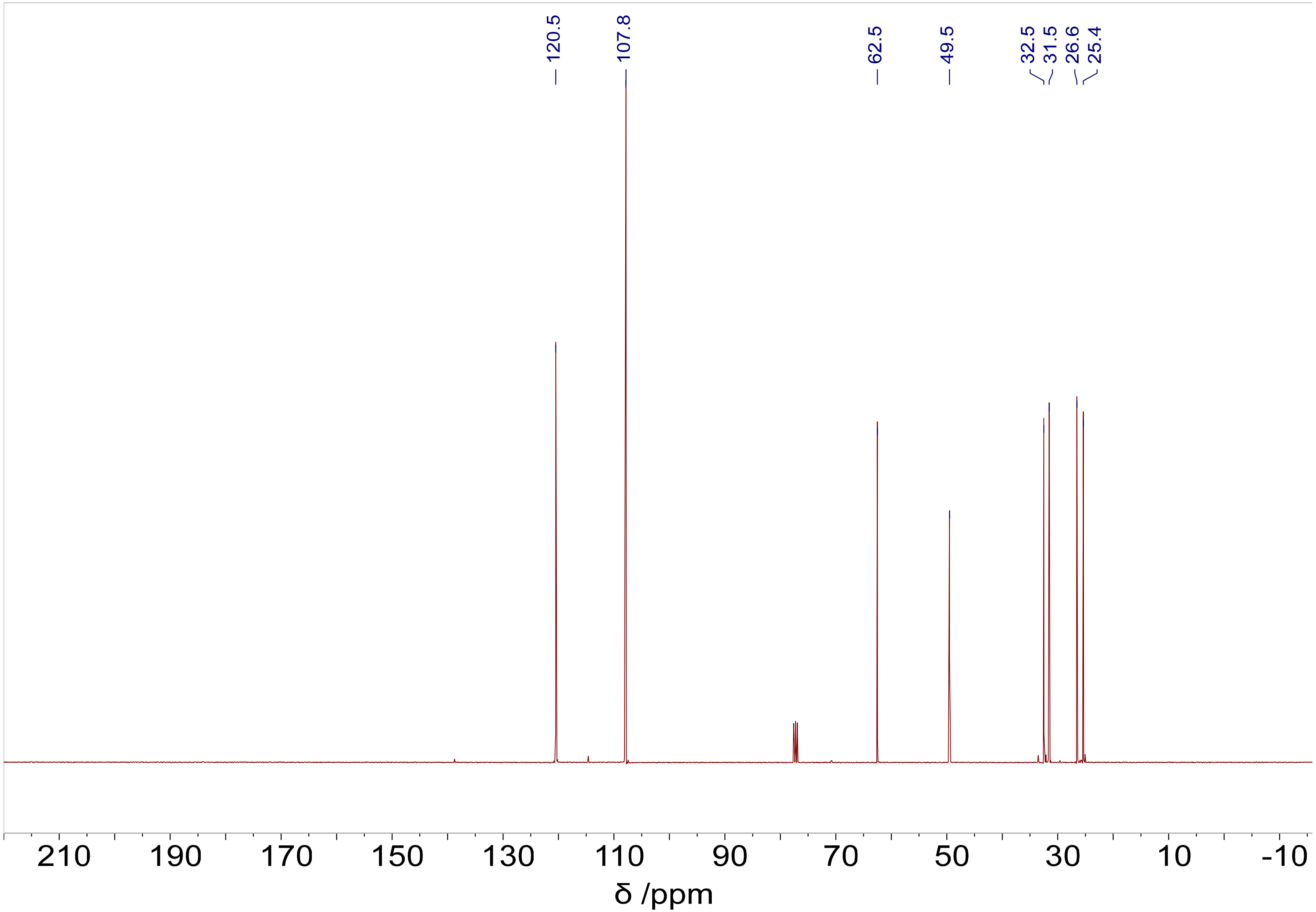

Figure S2. $^{13}C\{^1H\}$-NMR spectrum of 6-pyrrolylhexanol in $CDCl_3$.

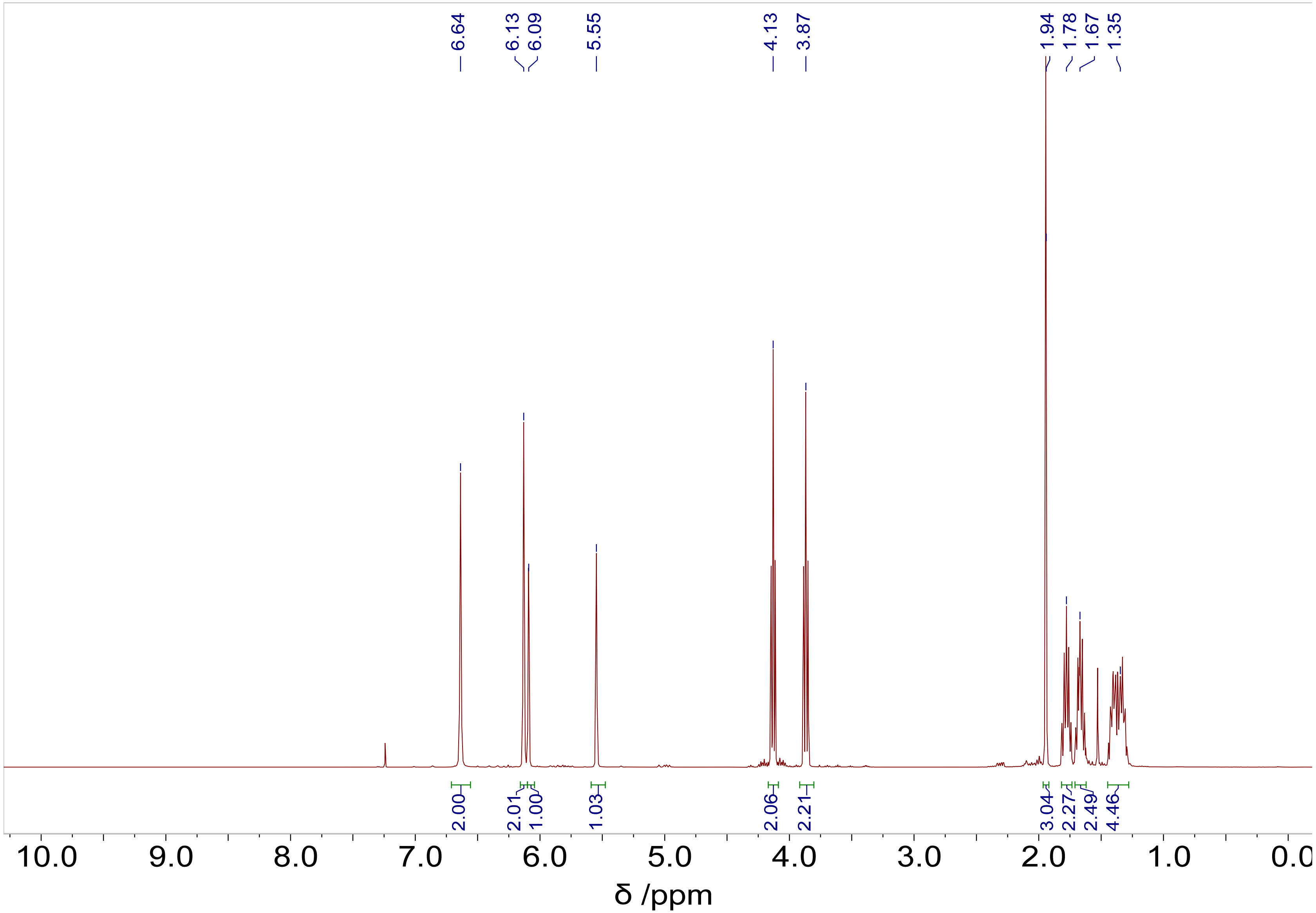

Figure S3. ^{1}H-NMR spectrum of 6-pyrrolylhexyl methacrylate in $CDCl_3$.

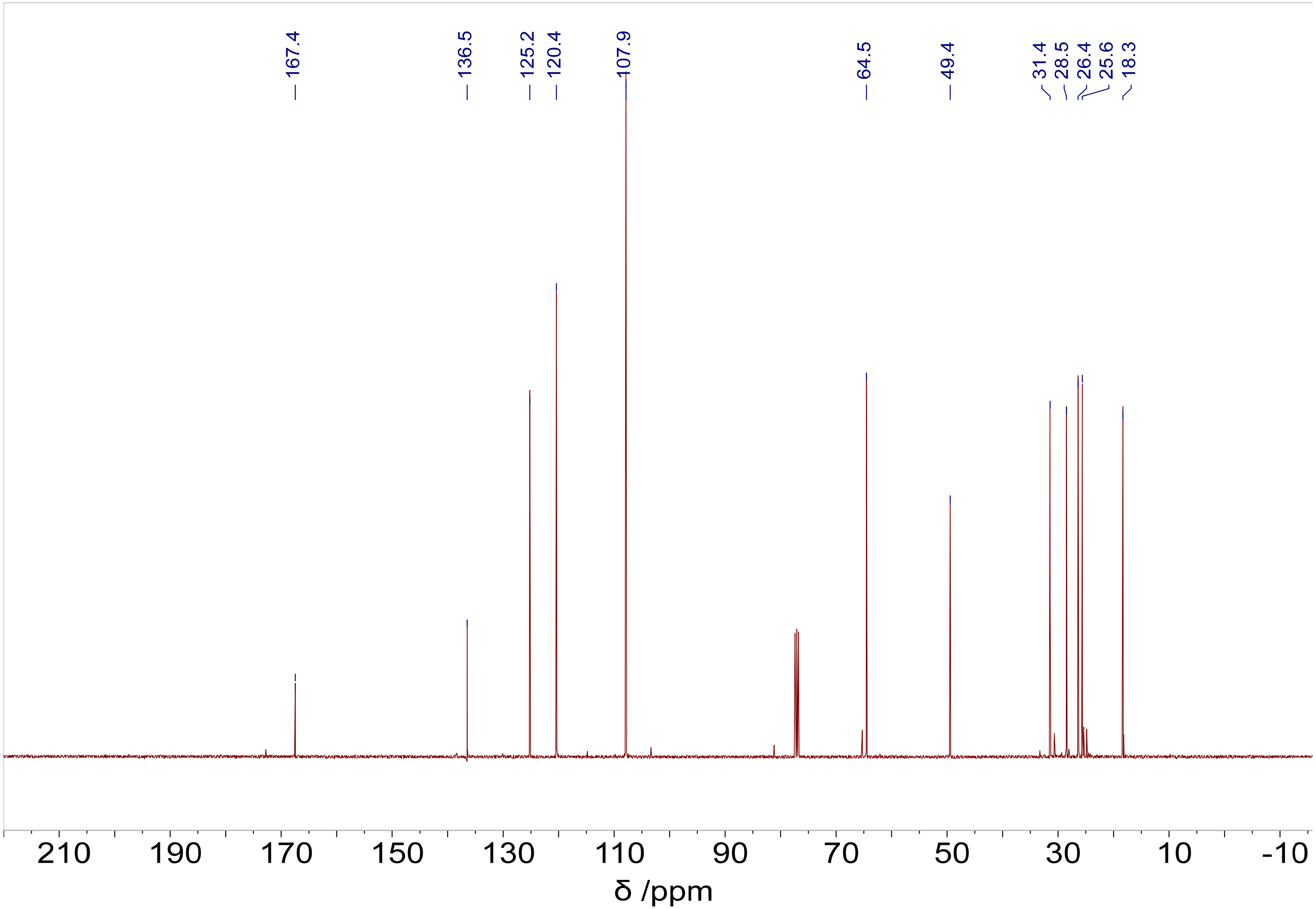

Figure S4. $^{13}C\{^{1}H\}$-NMR spectrum of 6-pyrrolylhexyl methacrylate in $CDCl_3$.

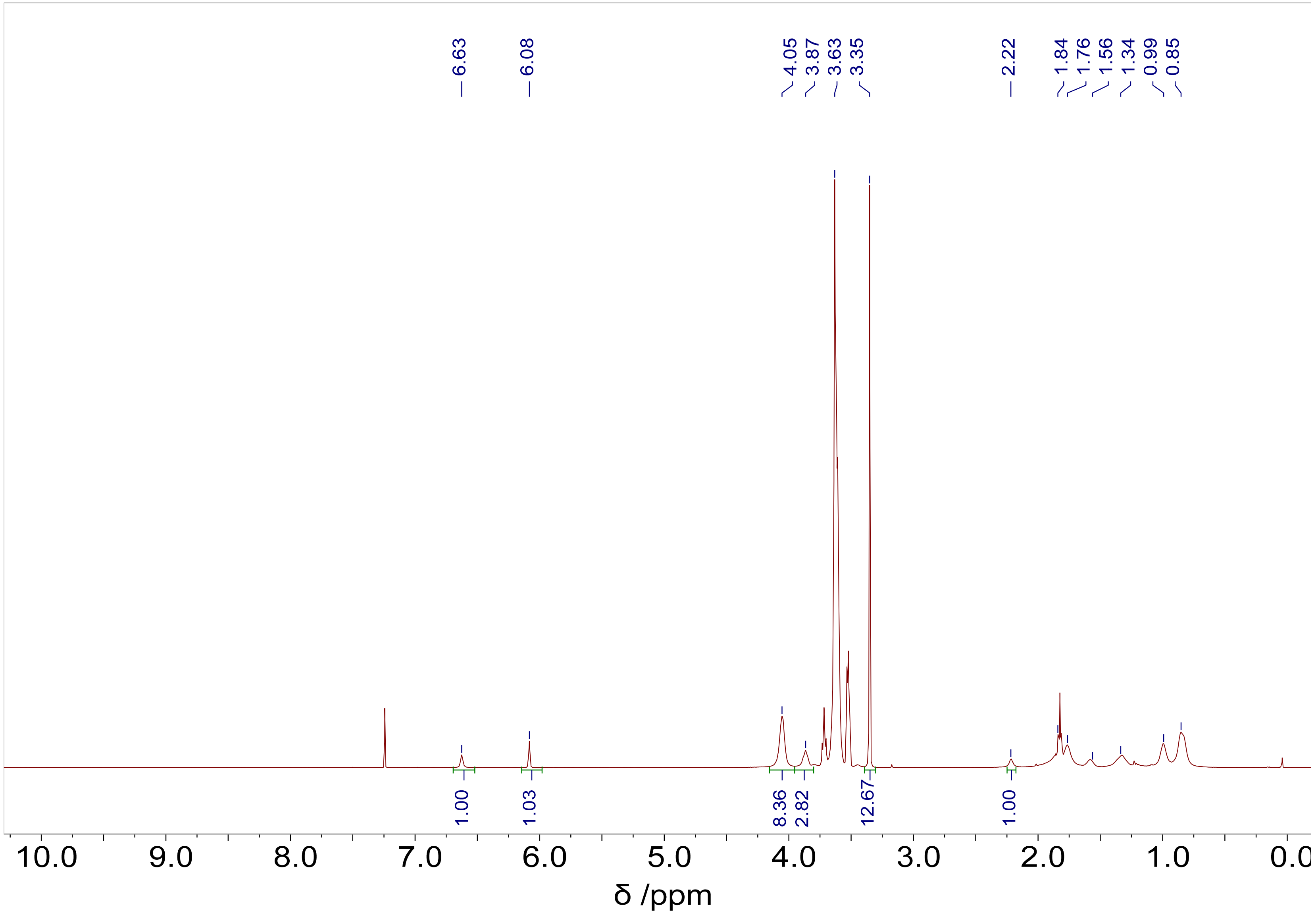

Figure S5. ^{1}H-NMR spectrum of the precursor polymer in $CDCl_3$.

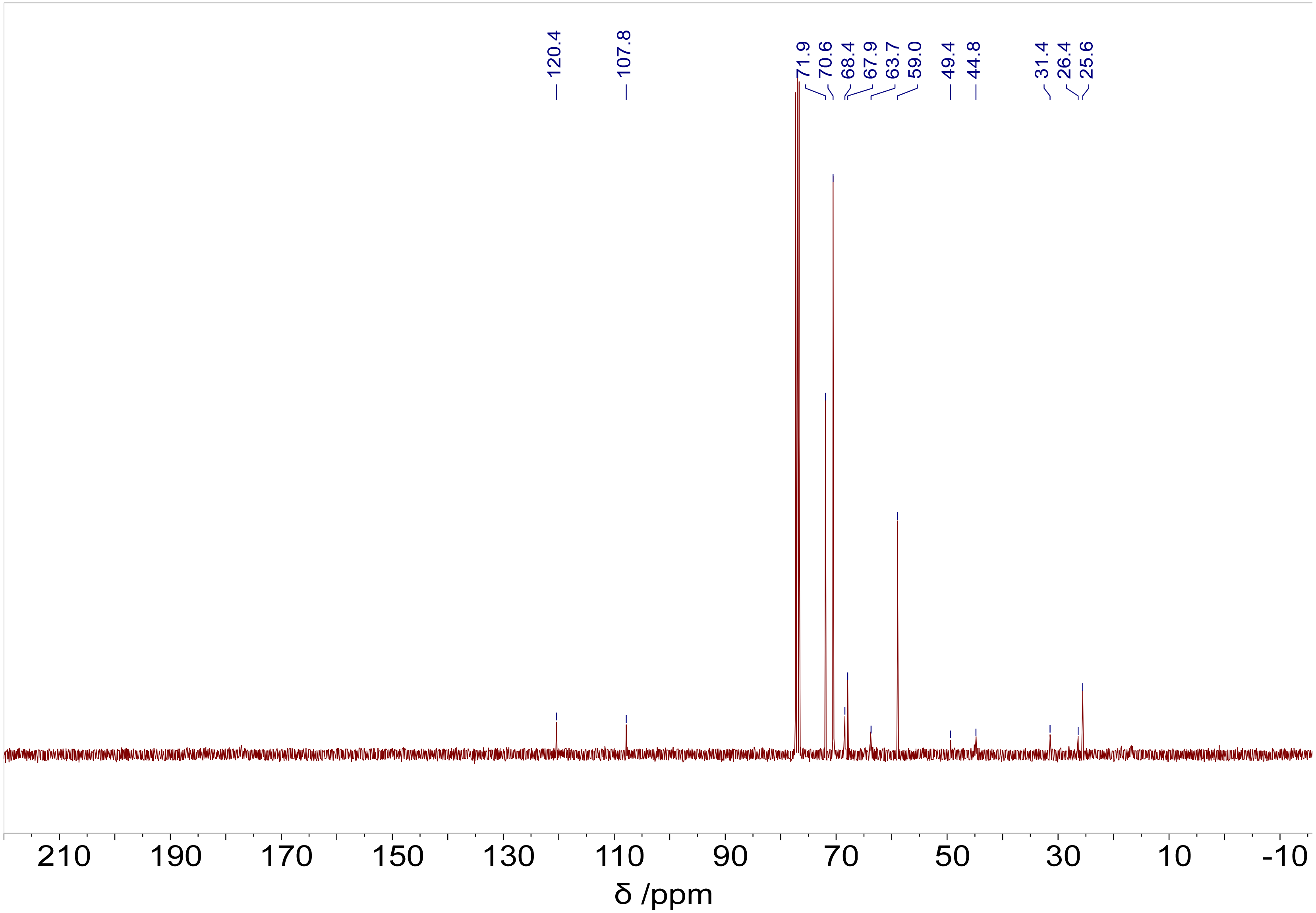

Figure S6. $^{13}C\{^{1}H\}$-NMR spectrum of the precursor polymer in $CDCl_3$.

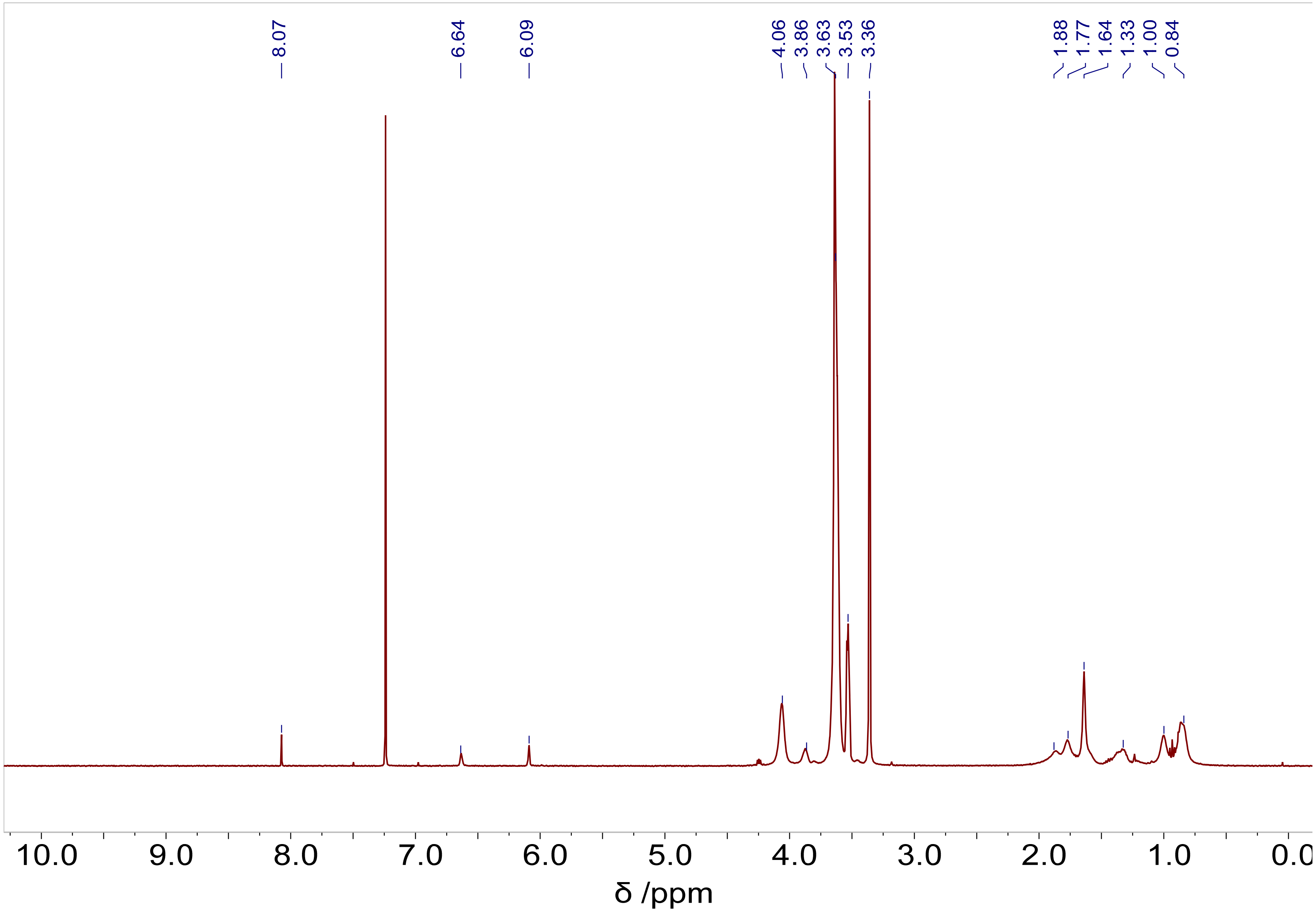

Figure S7. ^{1}H-NMR spectrum of $SCNP_2$ in $CDCl_3$.

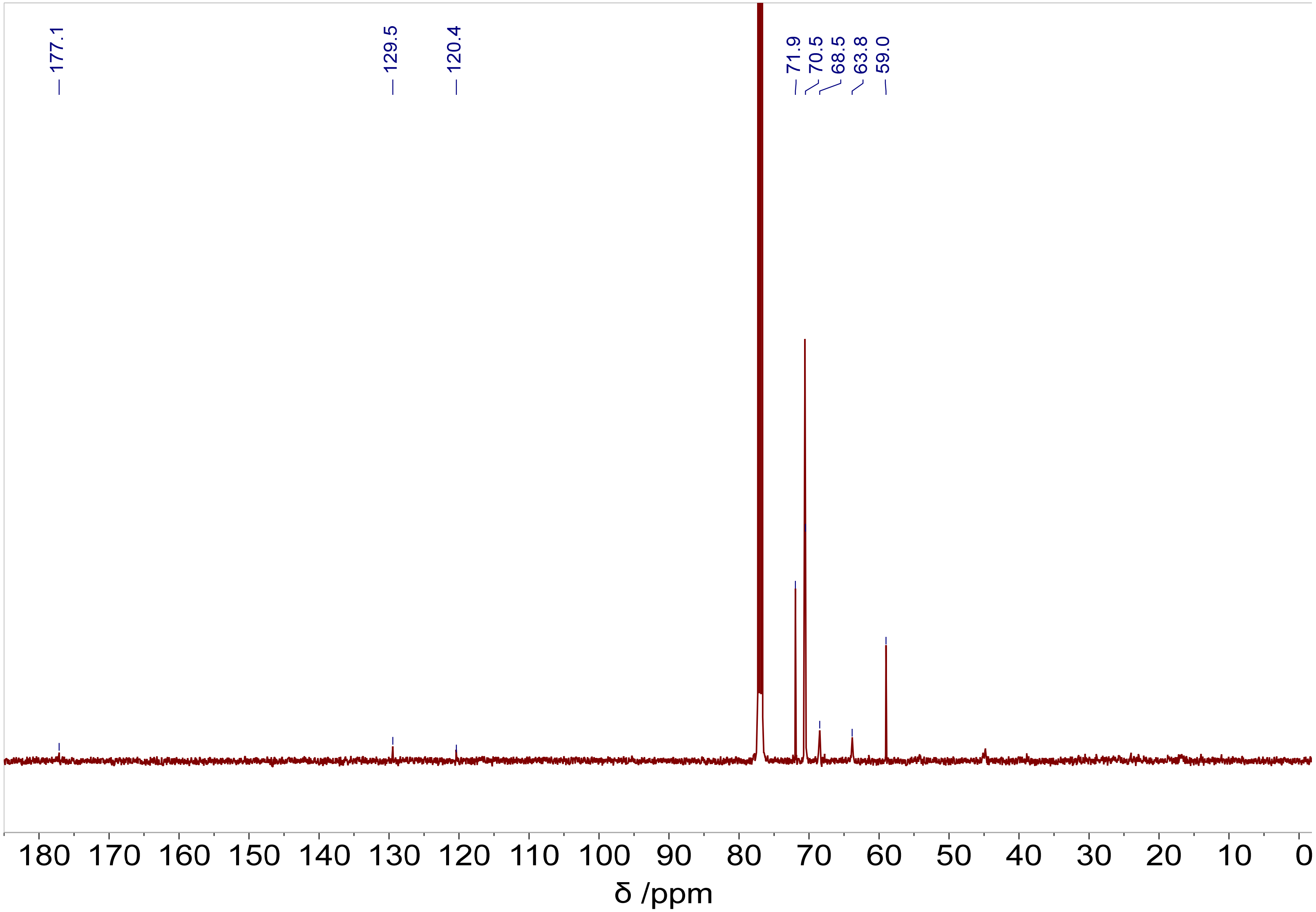

Figure S8. $^{13}C\{^1H\}$-NMR spectrum of $SCNP_2$ in $CDCl_3$.

Supporting Figures and Tables

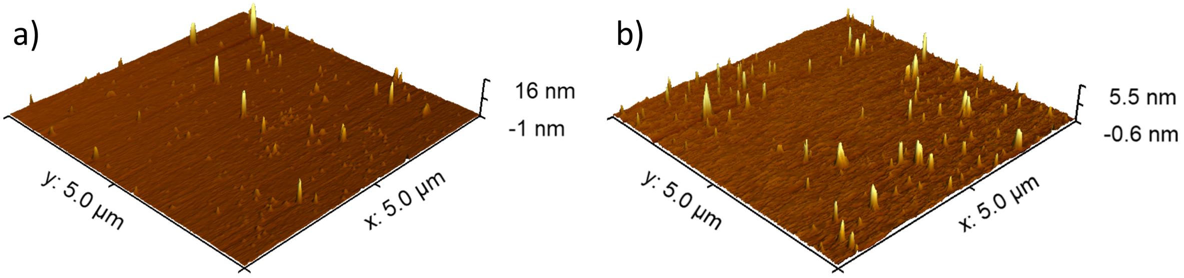

Figure S9. AFM height profiles of a) the precursor polymer and b) $SCNP_5$.

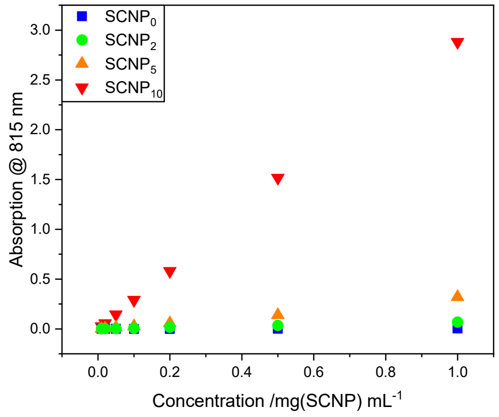

Figure S10. Lambert-Beer plots of the SCNPs in water at 815 nm based on the SCNP concentration.

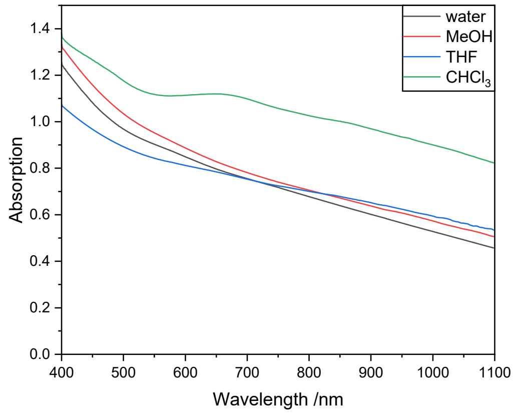

Figure S11. Absorption spectra of $SCNP_{10}$ in various solvents at concentrations of 0.1 mg/mL

Table S1. Temperature variation $T_{var,\ max}$ at different concentrations of $SCNP_{10}$ in water after 15 minutes irradiation using an NIR LED lamp (722 nm, 0.84 W/cm²). Weight-based efficiency and energy conversion estimated from the specific heat capacity of water.

SCNP concentration /mg mL^{-1}	PPy concentration /µg mL^{-1}	T_{var} /°C	Energy Conversion Efficiency a) /%
water	0	15.8 ± 0.1	25.7
0.1	20.4	40.2 ± 1.4	65.5
0.5	102.1	47.1 ± 1.7	76.7
1.0	204.2	45.7 ± 1.8	74.5

a) Estimated from T_{var}, the input energy (256.5 J), and the specific heat capacity of water (c_{H2O} = 4.18 J g^{-1} K^{-1}).

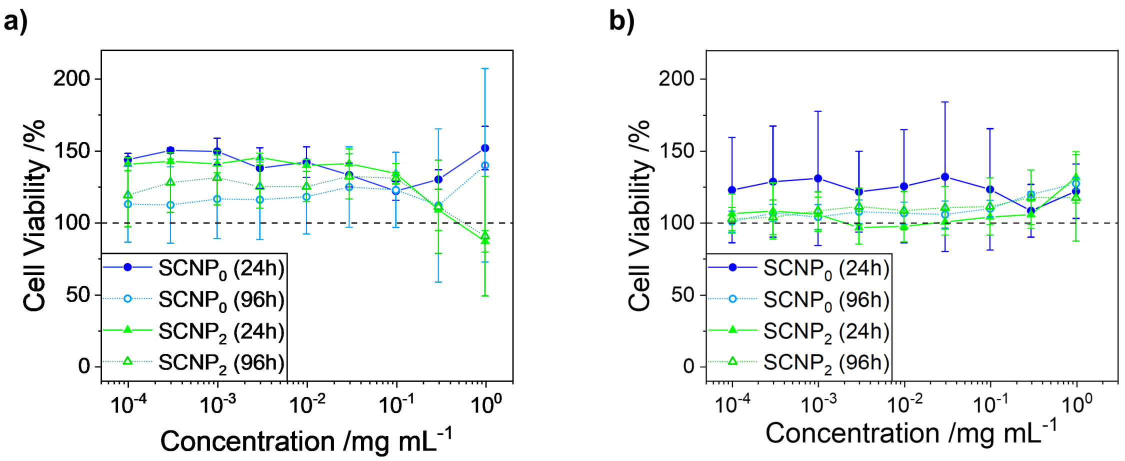

Figure S12. Cell viability assay of $SCNP_0$ and $SCNP_2$ on a) 3T3 and b) DLD-1 cell lines, incubated for 24h and 96h, respectively.

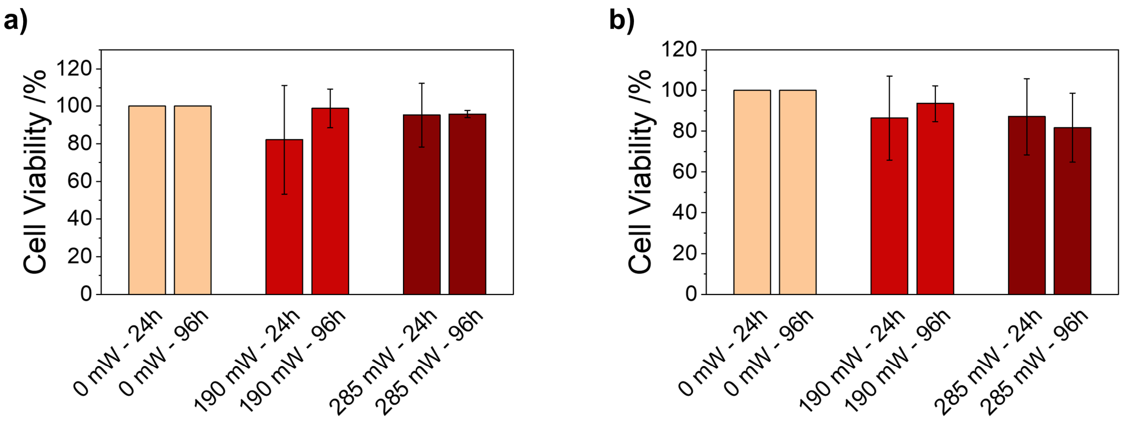

Figure S13. Phototoxicity assay of the NIR-LED array after illumination for 15 minutes of a) 3T3 cells and b) DLD-1 cells at different radiant fluxes per well and after different incubation times.

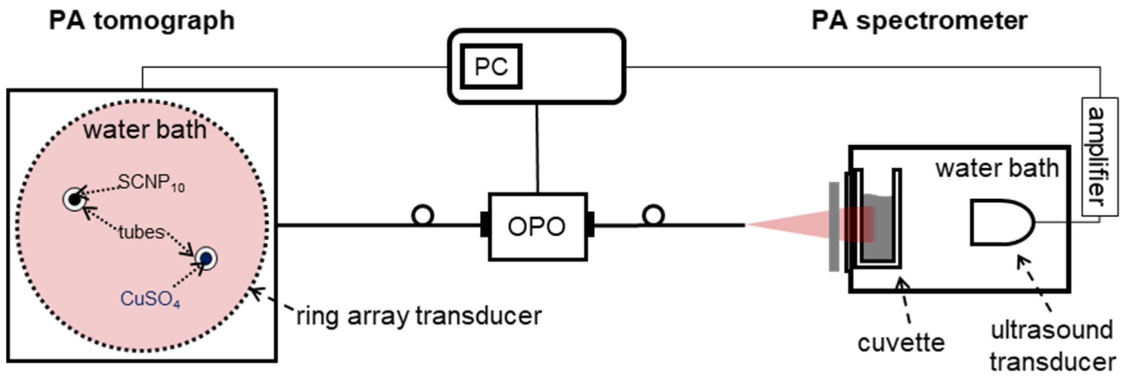

Figure S14. Schematic illustration of the PA spectrometer (right) and PA tomograph (left).
