# Supplementary material for: Photo-thermoresponsive polypyrrole-crosslinked single-chain nanoparticles for photothermal therapy
Source: Commun Chem. 2025 Apr 25;8:124. doi: 10.1038/s42004-025-01518-x (PMC12032120; doi:10.1038/s42004-025-01518-x)
Supplement: Supplementary file 3 — Reporting summary [file 42004_2025_1518_MOESM3_ESM.pdf]

nature portfolio

Corresponding author(s): Wolfgang H. Binder

Last updated by author(s): 01.04.2025

Reporting Summary

Nature Portfolio wishes to improve the reproducibility of the work that we publish. This form provides structure for consistency and transparency in reporting. For further information on Nature Portfolio policies, see our Editorial Policies and the Editorial Policy Checklist.

Statistics

For all statistical analyses, confirm that the following items are present in the figure legend, table legend, main text, or Methods section.

n/a	Confirmed	
☐	☒	The exact sample size (*n*) for each experimental group/condition, given as a discrete number and unit of measurement
☒	☐	A statement on whether measurements were taken from distinct samples or whether the same sample was measured repeatedly
☒	☐	The statistical test(s) used AND whether they are one- or two-sided<br>*Only common tests should be described solely by name; describe more complex techniques in the Methods section.*
☒	☐	A description of all covariates tested
☒	☐	A description of any assumptions or corrections, such as tests of normality and adjustment for multiple comparisons
☒	☐	A full description of the statistical parameters including central tendency (e.g. means) or other basic estimates (e.g. regression coefficient) AND variation (e.g. standard deviation) or associated estimates of uncertainty (e.g. confidence intervals)
☒	☐	For null hypothesis testing, the test statistic (e.g. *F*, *t*, *r*) with confidence intervals, effect sizes, degrees of freedom and *P* value noted<br>*Give P values as exact values whenever suitable.*
☒	☐	For Bayesian analysis, information on the choice of priors and Markov chain Monte Carlo settings
☒	☐	For hierarchical and complex designs, identification of the appropriate level for tests and full reporting of outcomes
☒	☐	Estimates of effect sizes (e.g. Cohen's *d*, Pearson's *r*), indicating how they were calculated

Our web collection on statistics for biologists contains articles on many of the points above.

Software and code

Policy information about availability of computer code

Data collection	In vitro cell viability assays were quatified using a Cytation5 Imaging Reader equipped with the Gen5 3.12 software.
Data analysis	Two different spreadsheet software tools were used for calculation of mean and SD as well es normalization on untreated controls: Libre Office Calc and Microsoft Office Excel.

For manuscripts utilizing custom algorithms or software that are central to the research but not yet described in published literature, software must be made available to editors and reviewers. We strongly encourage code deposition in a community repository (e.g. GitHub). See the Nature Portfolio guidelines for submitting code & software for further information.

Data

Policy information about availability of data

All manuscripts must include a data availability statement. This statement should provide the following information, where applicable:

- Accession codes, unique identifiers, or web links for publicly available datasets
- A description of any restrictions on data availability
- For clinical datasets or third party data, please ensure that the statement adheres to our policy

The evaluated data of this study are available within this article and its Supplementary Information. The raw data that support the findings of this study are provided to the publisher and are additionally stored electronically (eLabFTW) according to the requirements of the DFG.

Research involving human participants, their data, or biological material

Policy information about studies with human participants or human data. See also policy information about sex, gender (identity/presentation), and sexual orientation and race, ethnicity and racism.

Reporting on sex and gender	Only in vitro cell culture experiements were performed using commercially available cell lines.
Reporting on race, ethnicity, or other socially relevant groupings	Only in vitro cell culture experiements were performed using commercially available cell lines.
Population characteristics	Only in vitro cell culture experiements were performed using commercially available cell lines.
Recruitment	Only in vitro cell culture experiements were performed using commercially available cell lines.
Ethics oversight	Only in vitro cell culture experiements were performed using commercially available cell lines.

Note that full information on the approval of the study protocol must also be provided in the manuscript.

Field-specific reporting

Please select the one below that is the best fit for your research. If you are not sure, read the appropriate sections before making your selection.

☒ Life sciences ☐ Behavioural & social sciences ☐ Ecological, evolutionary & environmental sciences

For a reference copy of the document with all sections, see nature.com/documents/nr-reporting-summary-flat.pdf

Life sciences study design

All studies must disclose on these points even when the disclosure is negative.

Sample size	All in vitro cell vitality assays were perforemd on 96 well plates where each condition filled one column (n=8, mean +/- SD in % was calculated - adressing internal asssay spread/variability). The experiments were at least replicated 3 times independently (N=3, mean +/- SD in % was calculated - adressing reproducibilty)
Data exclusions	Only single data points were excluded where errors in the SOP occured, e.g. no Resazurin as vitality subtrate was added to a specific well in the 96 well plate etc. These were only rare singular events.
Replication	Cell culture experiments were performed at least in three independent replicates at different timepoints. Therefore, cell line passage dependency as well as daily procedure dependency etc. can be excluded.
Randomization	Blinding was not relevant. Only in vitro cell culture studies were performed.
Blinding	Blinding was not relevant. Only in vitro cell culture studies were performed.

Reporting for specific materials, systems and methods

We require information from authors about some types of materials, experimental systems and methods used in many studies. Here, indicate whether each material, system or method listed is relevant to your study. If you are not sure if a list item applies to your research, read the appropriate section before selecting a response.

Materials & experimental systems

n/a	Involved in the study	
☒	☐	Antibodies
☐	☒	Eukaryotic cell lines
☒	☐	Palaeontology and archaeology
☒	☐	Animals and other organisms
☒	☐	Clinical data
☒	☐	Dual use research of concern
☒	☐	Plants

Methods

n/a	Involved in the study	
☒	☐	ChIP-seq
☒	☐	Flow cytometry
☒	☐	MRI-based neuroimaging

Eukaryotic cell lines

Policy information about cell lines and Sex and Gender in Research

Cell line source(s)	Cell lines were purchased by either offical suppliers (DSMZ) or via a cooperation partner (here purchased from ATCC).
Authentication	None of the cell line was authenticated after purchasing it from the official supplier.
Mycoplasma contamination	All cell lines were tested negative for Mycoplasma contamination.
Commonly misidentified lines (See ICLAC register)	No commonly misidentfied line was used.

Plants

Seed stocks	*Report on the source of all seed stocks or other plant material used. If applicable, state the seed stock centre and catalogue number. If plant specimens were collected from the field, describe the collection location, date and sampling procedures.*
Novel plant genotypes	*Describe the methods by which all novel plant genotypes were produced. This includes those generated by transgenic approaches, gene editing, chemical/radiation-based mutagenesis and hybridization. For transgenic lines, describe the transformation method, the number of independent lines analyzed and the generation upon which experiments were performed. For gene-edited lines, describe the editor used, the endogenous sequence targeted for editing, the targeting guide RNA sequence (if applicable) and how the editor was applied.*
Authentication	*Describe any authentication procedures for each seed stock used or novel genotype generated. Describe any experiments used to assess the effect of a mutation and, where applicable, how potential secondary effects (e.g. second site T-DNA insertions, mosiacism, off-target gene editing) were examined.*
